# Efficacy of ICI-based treatment in advanced NSCLC patients with PD-L1≥50% who developed EGFR-TKI resistance

**DOI:** 10.3389/fimmu.2023.1161718

**Published:** 2023-05-17

**Authors:** Yujing Li, Haohua Jiang, Fangfei Qian, Ya Chen, Wensheng Zhou, Yanwei Zhang, Jun Lu, Yuqing Lou, Baohui Han, Wei Zhang

**Affiliations:** ^1^ Department of Respiratory and Critical Care Medicine, Shanghai Chest Hospital, Shanghai Jiao Tong University School of Medicine, Shanghai, China; ^2^ Department of Respiratory and Critical Care Medicine, The First Affiliated Hospital of University of Science and Technology (USTC), Division of Life Science and Medicine, University of Science and Technology of China, Hefei, China; ^3^ State Key Laboratory of Respiratory Disease, National Clinical Research Center for Respiratory Disease, Guangzhou Institute of Respiratory Health, The First Affiliated Hospital of Guangzhou Medical University, Guangzhou, China

**Keywords:** non-small-cell lung cancer, immunotherapy, drug resistance, epidermal growth factor receptor-tyrosine kinase inhibitor (EGFR-TKI), programmed death ligand 1 (PD-L1)

## Abstract

**Introduction:**

Platinum-based chemotherapy is still the standard of care for Epidermal growth factor receptor (EGFR) mutated non-small cell lung cancer (NSCLC) patients after developing EGFR-TKI resistance. However, no study focusing on the role of immuno checkpoint inhibitor (ICI) based treatments for EGFR mutated NSCLC patients who carried programmed death ligand 1 (PD-L1) tumor proportion score (TPS) greater than 50% progressed after EGFR-TKI therapy. In this study, we retrospectively investigated the outcomes of ICI-based treatments for EGFR mutated NSCLC patients carried PD-L1 TPS≥50% after developing EGFR-TKI resistance and to explore the population that may benefited from ICI-based treatment.

**Methods:**

We retrospectively collected data of advanced NSCLC patients with EGFR mutations and PD-L1 TPS≥50% who have failed prior EGFR-TKI therapies without T790M mutation at Shanghai Chest Hospital between January 2018 and June 2021. Progression-free survival (PFS) and overall survival (OS) were utilized to evaluate the outcomes of this study.

**Results:**

A total of 146 patients were included. Up to June 20th, 2022, median follow-up was 36.7 months (IQR, 12.5-44.2 months). Among the population, 66 patients (45.2%) received chemotherapy, the remaning (54.8%) received ICI-based treatment, including 56 patients(70.0%) received ICI combined with chemotherapy (IC) and 24 patients (30.0%) received ICI monotherapy (IM). In IC group,31 patients received ICI combined with chemotherapy,19 patients received ICI combined with antiangiogenic therapy and remaing received ICI combined with chemotherapy and antiangiogenic therapy. Survival analysis shown that patients who received ICI-based treatment had better progress-free survival (PFS) and overall survival (OS) compared with those treated with other therapy (median PFS, 10.0 vs. 4.0 months, P<0.001; median OS, 39.5 vs. 24.2 months, P<0.001). What’s more, patients who treated with IC treatment had a superior survival time than those received IM treatment (median PFS, 10.3 vs. 7.0 months, P<0.001; median OS, 41.6 vs. 32.4 months, P<0.001). Subgroup analysis found that the PFS and OS benefit of IC was evident in all subgroups.

**Conclusions:**

For advanced NSCLC patients with EGFR mutations and PD-L1 TPS≥50% who have failed prior EGFR-TKI therapies without T790M mutation, ICI-based treatment could provide a more favorable survival than classical chemotherapy. What’ s more, compared with ICI monotherapy, ICI combined with chemotherapy seems to be the preferred treatment.

## Introduction

Lung cancer remains the most prevalent malignancy worldwide, with non-small cell lung cancer (NSCLC) accounting for approximately 85% of all newly diagnosed lung cancers (1, 2). For patients with advanced NSCLC harboring epidermal growth factor receptor (EGFR) mutations, EGFR-tyrosine kinase inhibitors (EGFR-TKIs) are usually considered the first-line treatment (3–5). However, drug-acquired resistance is inevitable. Platinum-based chemotherapy remains the standard of care for patients with non-small cell lung cancer (NSCLC) with epidermal growth factor receptor (EGFR) mutations after developing EGFR-TKI resistance without EGFR T790M mutation, while the clinical benefit was limited (6).

In recent years, immune checkpoint inhibitors have dramatically changed the standard of care for patients with advanced NSCLC. Nevertheless, the response to immunotherapy seems to vary depending on the inherent immune microenvironment (7, 8). For example, NSCLC patients with PD-L1 tumor proportion score (TPS) ≥ 50% seem to benefit from immunotherapy, but for those carrying EGFR-sensitive mutations and ALK rearrangements (EGFR+/ALK+), the response to immunotherapy appears to be poor.

Few studies have investigated second-line treatment strategies for EGFR-mutant NSCLC patients carrying PD-L1 TPS greater than 50% who progressed after EGFR-TKI therapy. The possible reason for this is that EGFR-mutated NSCLC usually has a lower level of PD-L1 expression (9, 10), and NSCLC patients carrying EGFR mutations with PD-L1 TPS greater than 50% account for approximately 11.8% of all non-small cell lung cancers. In this study, we retrospectively investigated the outcome of NSCLC patients with EGFR mutations carrying PD-L1 TPS ≥ 50% after developing EGFR-TKI resistance with ICI therapy and explored the population that may benefit from ICI therapy.

## Materials and methods

### Study design and patients

We retrospectively collected 2037 patients carrying EGFR mutations treated at Shanghai Chest Hospital between January 2018 and June 2021 and identified them from the database. Our inclusion criteria indluding (1): diagnosed with non-small cell lung cancer; (2) carry EGFR mutations; (3) receive EGFR-TKI as first line treatment. Some of these patients were excluded according to the following criteria: (1) other driver mutations; (2) any recent surgery; (3) negative PD-L1 expression or PD-L1 TPS < 50%; (4) diagnosis of other tumors; (5) incomplete clinical information; (6) missed follow-up; (7) receiving chemotherapy or immunotherapy in first-line treatment and (8) carry T790M mutation after developing EGFR-TKI resistance. Also, clinicopathological characteristics such as gender, age, TNM stage, smoking history, histology, and treatment details were recorded. This study was approved by the Institutional Review Board of the Shanghai Chest Hospital and was conducted following the Declaration of Helsinki.

### Detection of genes and PD-L1 TPS

Tissue samples were obtained at disease diagnosis before first-line treatment or after developing EGFR-TKI resistance, and EGFR mutations were detected by next-generation sequencing (NGS) or single-gene test (LungCureCDx, Burning Rock, Suzhou, China). Assessment of PD-L1 expression before first-line therapy or or after developing EGFR-TKI resistance by PD-L1 IHC 22C3 pharmDx assay (Agilent Technologies China, Beijing, China)

### Assessment and treatment

According to the International Association for the Study of Lung Cancer (IASLC) 8th edition tumor-node-metastasis (TNM) classification, the clinical stage was determined at the time of disease diagnosis. High-resolution chest computed tomography (HRCT) and abdominal ultrasound scans were performed every 6-8 weeks after treatment initiation to assess tumor response. For patients without brain metastases at baseline or without associated symptoms after that, brain magnetic resonance imaging (MRI) was performed every six months. Tumor response was assessed according to the Response Evaluation Criteria in Solid Tumors (RECIST version 1.1).

Experienced physicians completed all evaluations, and therapeutic schedules were decided and adjusted according to the patient’s condition and disease progression (including chemotherapy, anti-angiogenesis treatment, immunotherapy and their combinations).

### Follow up

Patients’ follow-up data were obtained from regular clinical records. Patients receiving chemotherapy or immunotherapy would be admitted monthly, while other outpatients were required to follow up at least every two months. Telephone interviews were also used to verify the information and to contact patients who were not followed up regularly. The primary endpoints of this study were PFS (from initiation of immunotherapy to disease progression or death; if patients do not receive PD-1 inhibitors, then d0 should be the start of second-line therapy) and OS (from initiation of immunotherapy to death or last follow-up). If the patient died, a date was used as the last follow-up.

### Statistical analysis

Categorical variables were compared using the Chi-square and Fisher’s exact test (percentage calculated). Median PFS and median OS, and between-group survival differences were determined using the Kaplan-Meier (KM) method and the Log-rank test. Univariate and multivariate analyses were performed using Cox proportional hazards models for significant independent risk factors for PFS and OS. Factors with P < 0.2 in univariate analysis were further incorporated into the multivariate analysis. All P values were two-sided, and statistically significant differences were considered when p < 0.05. All statistical analyses were performed using SPSS version 28.0 (IBM Corporation, Armonk, NY, USA) and R software (version 4.0.2, R Foundation for Statistical Computing, Vienna, Austria).

## Results

### Patient characteristics

**Figure 1:**
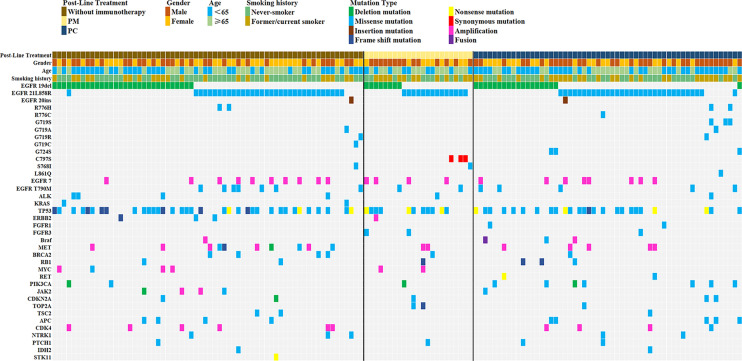
Molecular features of the EGFR-mutated NSCLC patients with PD-L1≥50% who developed EGFR-TKI resistance.

After screening, 146 patients met the above criteria and were divided into three groups. Patients received either chemotherapy(n=32), anti-angiogenesis(n=11) or both(n=13) were included in the immunotherapy negative (IN) group. Similarly, patients in the IM group received ICI monotherapy (n = 24, 16.4%), and in the IC group received both immunotherapy and anti-angiogenic therapy or chemotherapy (n = 56, 38.35%, **Figure 1**). Complete baseline characteristics of both groups are shown in **Table 1**. 78 (53.4%) patients were male, 68 (46.4%) were female, 81 patients were under 65 years of age (55.5%), and most of them were stage IV (91.1%). In addition, 62 (42.5%) were former or current smokers. All variables were balanced between the two groups and did not differ statistically (p > 0.05).

**Table 1:** Clinical characteristics for all patients.

Characteristics	Total cohort (n=146) (%)	Immunotherapy		*P* value
		Without (n=66) (%)	With (n=80) (%)	
**Gender**				0.080
**Male**	78 (53.4)	30 (45.5)	48 (60.0)	
**Female**	68 (46.6)	36 (54.5)	32 (40.0)	
**Age(y)**				0.898
**<65**	81 (55.5)	37 (56.1)	44 (55.0)	
**≥65**	65 (44.5)	29 (43.9)	36 (45.0)	
**Smoking History**				0.091
**Never-smoker**	84 (57.5)	43 (65.2)	41 (51.2)	
**Former/current smoker**	62 (42.5)	23 (34.8)	39 (48.8)	
**TNM stage**				0.215
**III**	13 (8.9)	8 (12.1)	5 (6.3)	
**IV**	133 (91.1)	58 (87.9)	75 (93.7)	
**Histology**				0.196
**Squamous**	2 (1.4)	0 (0.0)	2 (2.5)	
**Adenocarcinoma**	144 (98.6)	66 (100.0)	78 (97.5)	
**ECOG-PS**				0.472
**0-1**	136 (93.2)	63 (95.5)	73 (91.2)	
**2**	10 (6.8)	3 (4.5)	7 (8.8)	
**EGFR mutation type**				0.298
**19del**	59 (40.4)	31 (47.0)	28 (35.0)	
**21L858R**	75 (51.4)	31 (47.0)	44 (55.0)	
**Otders**	12 (8.2)	4 (6.0)	8 (10.0)	
**Primary brain metastasis**				0.210
**Yes**	43 (29.5)	16 (19.4)	27 (23.6)	
**No**	103 (70.5)	50 (46.6)	53 (56.4)	
**Primary liver metastasis**				0.517
**Yes**	11 (7.5)	6 (9.1)	5 (6.3)	
**No**	135 (92.5)	60 (90.9)	75 (93.7)	
**EGFR-TKI**				0.358
**Gefitinib**	51 (34.9)	21 (31.9)	30 (37.5)	
**Icotinib**	53 (36.3)	22 (33.3)	31 (38.8)	
**Erlotinib**	7 (4.8)	5 (7.6)	2 (2.5)	
**Afatinib**	10 (6.8)	4 (6.1)	6 (7.5)	
**Osimertinib**	23 (15.8)	13 (19.6)	10 (12.5)	
**Dacomitinib**	2 (1.4)	1 (1.5)	1 (1.2)	

Pathological specimens from all patients were tested for EGFR mutations by single-gene test or NGS. 58 (39.7%) patients had EGFR exon 19 deletions, 75 (51.4%) patients had EGFR exon 21 L858R mutations, 13(8.9%) patients carried EGFR T790M mutation and 20 (13.7%) patients had other rare EGFR mutations, such as S768I missense mutation (n=2), C797S cis-mutation (n=3), exon 20ins (n=2), R776X missense mutation (n=5), G719X missense mutation (n=8), G724S missense mutation (n=2) and L861Q missense mutation (N=1). Incidentally, the most common combined mutation was TP53 (n = 72, 49.32%), and various missense mutations (n = 53, 73.61%) were most common among TP53 mutations (**Figure 1**).

### Survival analysis

Until June 20th, 2022, the median follow-up time was 36.7 months (IQR, 12.5-44.2 months). Among a total of 146 patients, tumor progression occurred in all patients. 43 (29.5%) patients had brain metastasis, and 16 (11.0%) patients had liver metastasis. Most recurrent sites were in the lungs (42.86%), bones (15.07%), and brain (11.64%).

**Figure 2:**
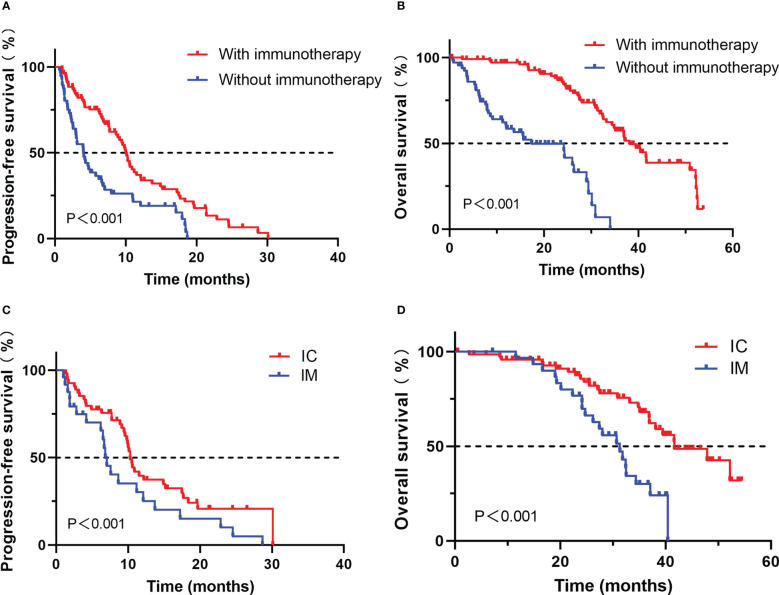
Comparison of progression-free survival **(A)** and overall survival **(B)** for advanced NSCLC patients with PD-L1≥50% who developed EGFR-TKI resistance treated with or without immunotherapy; Comparison of progression-free survival **(C)** and overall survival **(D)** for advanced NSCLC patients with PD-L1≥50% who developed EGFR-TKI resistance treated with IC or IM.

Survival analysis showed that patients treated with ICIs had better progression-free survival (PFS) and overall survival (OS) compared with those treated with other treatments (median PFS, 10.0 vs. 4.0 months, P < 0.001; median OS, 39.5 vs. 24.2 months, P < 0.001, **Figure 2**).

Factors affecting PFS and OS were enrolled (**Tables 2**, **3**). Cox proportional-hazards models were used to analyze the factors that might impact PFS and OS. P < 0.2 was considered significant in the univariable analysis. In the univariate analysis, we found that ECOG PS state, EGFR mutation type, primary liver metastasis, and post-line immunotherapy were significant factors affecting PFS (p < 0.001, p = 0.120, p = 0.038, and p < 0.001, respectively) to improve sensitivity. These variables were further incorporated into the multivariate analysis, which showed that poor PS state, primary liver metastasis, and absence of immunotherapy were independent predictors of PFS (p < 0.001, p = 0.044, p < 0.001, respectively; **Table 2**). In terms of OS, univariate analysis revealed that age, ECOG PS state, primary liver metastasis, and post-line immunotherapy were significant factors for OS (p = 0.122, p = 0.006, p = 0.032, p = 0.012, respectively). Further multivariate analysis showed that all these variables were also independent risk factors for OS (p = 0.008, p = 0.037, p = 0.005, respectively; **Table 3**).

**Table 2:** Univariable and multivariable analysis for progression-free survival (PFS) in all patients.

Characteristics	Univariable analysis			Multivariable analysis		
	HR	95%Cl	*P*	HR	95%Cl	*P*
**Gender**			0.416			
**Male**	reference					
**Female**	0.852	0.579-1.254				
**Age(y)**			0.740			
**<65**	reference					
**≥65**	0.937	0.640-1.373				
**Smoking History**			0.315			
**Yes**	reference					
**No**	0.892	0.830-1.782				
**TNM stage**			0.948			
**III**	reference					
**IV**	1.021	0.544-1.917				
**Histology**			0.676			
**Squamous**	reference					
**Adenocarcinoma**	0.741	0.182-3.019				
**ECOG-PS**			**<0.001**			**<0.001**
**0-1**	reference			reference		
**2**	5.675	2.544-7.658		5.363	2.376-12.106	
**EGFR mutation type**			0.120			0.363
**19del**	reference			reference		
**21L858R**	0.764	0.509-1.145	0.192	0.329	0.547-1.257	0.377
**Otders**	0.908	0.453-1.821	0.074	1.312	0.638-2.699	0.461
**Primary brain metastasis**			0.901			
**Yes**	reference					
**No**	1.027	0.673-1.569				
**Primary liver metastasis**			**0.038**			**0.044**
**Yes**	reference			reference		
**No**	0.481	0.241-0.959		0.572	0.278-0.902	
**Post-line immunotderapy**			**<0.001**			**<0.001**
**Yes**	reference			reference		
**No**	2.183	1.465-3.253		2.201	1.460-3.318	

ECOG-PS, Eastern cooperative oncology group-performance status; EGFR, epidermal growth factor receptor.

The bold values mean these characters are both significant in univariable and multivarible analysis.

**Table 3:** Univariable and multivariable cox regression analysis for overall survival (OS) in all patients.

Characteristics	Univariable analysis			Multivariable analysis		
	HR	95%Cl	*P*	HR	95%Cl	*P*
**Gender**			0.322			
**Male**	reference					
**Female**	0.767	0.454-1.297				
**Age(y)**			0.122			0.130
**<65**	reference			reference		
**≥65**	1.524	0.894-2.600		1.519	0.885-2.610	
**Smoking History**			0.734			
**Yes**	reference					
**No**	0.915	0.548-1.528				
**TNM stage**			0.400			
**III**	reference					
**IV**	1.441	0.616-3.372				
**Histology**			0.368			
**Squamous**	reference					
**Adenocarcinoma**	0.377	0.195-1.665				
**ECOG-PS**			**0.006**			**0.008**
**0-1**	reference			reference		
**2**	2.210	1.191-3.841		2.270	1.112-3.877	
**EGFR mutation type**			0.531			
**19del**	reference					
**21L858R**	0.789	0.452-1.379	0.406			
**Otders**	1.231	0.502-3.015	0.650			
**Primary brain metastasis**			0.745			
**Yes**	reference					
**No**	0.912	0.522-1.592				
**Primary liver metastasis**			**0.032**			**0.037**
**Yes**	reference			reference		
**No**	0.457	0.194-0.772		0.550	0.230-0.793	
**Post-line immunotderapy**			**0.012**			**0.005**
**Yes**	reference			reference		
**No**	1.963	1.163-3.314		2.184	1.273-3.746	

ECOG-PS, Eastern cooperative oncology group-performance status; EGFR, epidermal growth factor receptor.

The bold values mean these characters are both significant in univariable and multivarible analysis.

### Immunotherapy

**Figure 3:**
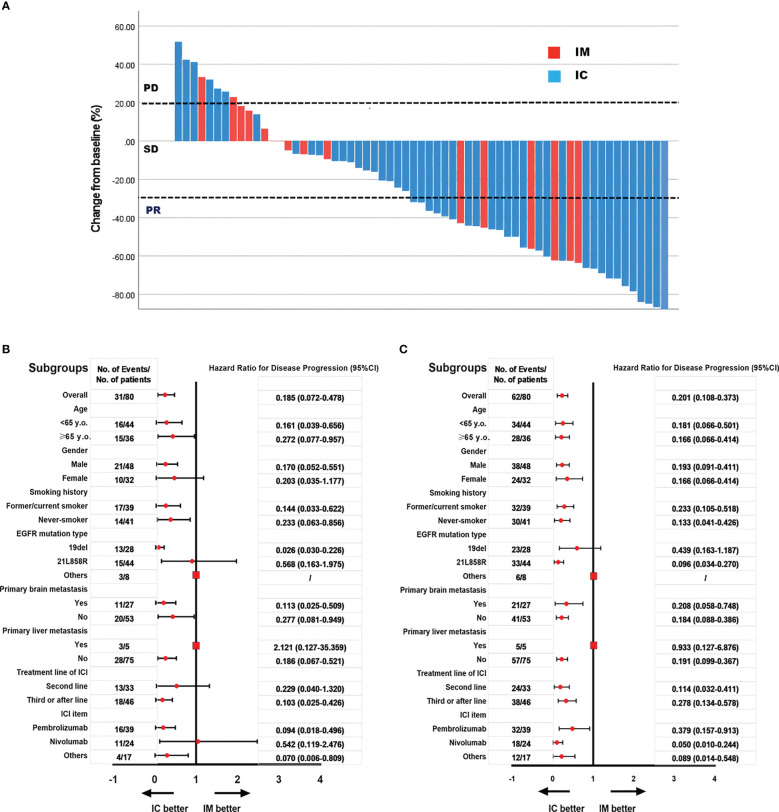
The objective response rate is shown as a percent change of target lesions from baseline in IC and IM groups **(A)**; Subgroups analysis of PFS in IC and IM groups **(B)**; Subgroups analysis of OS in IC and IM groups **(C)**.

We further analyzed the differences between the IC and IM groups (n = 80). All variants were balanced between IM and IC patients, except for physicians’ preference to use combination therapy in second-line treatment rather than further treatment (p = 0.01, **Table 4**). The objective response rate to immunotherapy reached 41.3% (n = 33), with 39 patients (48.2%) having stable disease and eight patients (11.0%) having progressive disease (**Figure 3A**).

**Table 4:** Clinical characteristics for patients with immunotherapy.

Characteristics	Total (n=80) (%)	Immunotherapy (n=80)		*P* value
		IM(n=24)(%)	IC (n=56) (%)	
**Gender**				0.765
**Male**	48(60)	15(62.5)	33(58.9)	
**Female**	32(40)	9(37.5)	23(41.1)	
**Age(y)**				0.117
**<65**	44(55)	10(41.7)	34(60.7)	
**≥65**	36(45)	14(58.3)	22(39.3)	
**Smoking History**				0.526
**Never-smoker**	41(51.2)	11(45.8)	30(53.6)	
**Former/current smoker**	39(48.8)	13(54.2)	26(46.4)	
**TNM stage**				0.131
**III**	5(6.2)	0(0)	5(8.9)	
**IV**	75(93.8)	24(100.0)	51(91.1)	
**Histologgy**				/
**Squamous**	0	0	0	
**Adenocarcinoma**	80(100)	24(100)	56(100)	
**ECOG-PS**				0.370
**0-1**	76(95.0)	22(91.7)	54(96.4)	
**2**	4(5.0)	2(8.3)	2(3.6)	
**EGFR mutation type**				0.523
**19del**	28(35.0)	9(37.5)	19(33.9)	
**21L858R**	44(55.0)	14(58.3)	30(53.6)	
**Otders**	8(10.0)	1(4.2)	7(12.5)	
**Primary brain metastasis**				0.327
**Yes**	27(33.8)	10(41.7)	17(30.4)	
**No**	53(66.3)	14(58.3)	39(69.6)	
**Primary liver metastasis**				0.131
**Yes**	5(6.2)	3(12.5)	2(3.6)	
**No**	75(93.8)	21(87.5)	54(96.4)	
**Treatment line of immunotderapy**				**0.010**
**Second line**	34(42.5)	5(20.8)	29(51.8)	
**Third or after line**	46(57.5)	19(79.2)	27(48.2)	
**Immunotderapy**				0.389
**Pembrolizumab**	39(48.8)	12(50.0)	27(48.2)	
**Nivolumab**	24(30.0)	9(37.5)	15(26.8)	
**Otders**	17(21.2)	3(12.5)	14(25.0)	

ECOG-PS, Eastern cooperative oncology group-performance status; EGFR, epidermal growth factor receptor.

The bold values mean these characters are both significant in univariable and multivarible analysis.

In our study, subgroup analysis revealed that the PFS and OS benefit of IC was significant in most subgroups, except for patients with primary liver metastases and other mutations of EGFR, because the sample was too small to calculate HR and 95% CI (**Figures 3B, C**).

### Change in PD-L1 expression

**Figure 4:**
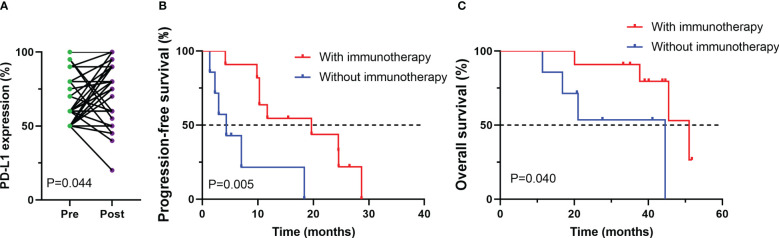
Changes in PD-L1 expression between before (pre) and after (post) EGFR-TKI treatment for patients with available paired tumor samples **(A)**; Progression-free survival (PFS) **(B)** and overall survival (OS) **(C)** among patients whose PD-L1 expression improved after developing EGFR-TKI resistance.

Among 38 patients who underwent PD-L1 immunohistochemical testing after developing EGFR-TKI resistance, we also explored changes in PD-L1 expression in tumor cells between before receiving EGFR-TKI treatment and the development of drug resistance. PD-L1 expression was remarkablely increased after receiving EGFR-TKI treatment (p=0.044, **Figure 4A**). Then, association of PD-L1 expression postprogression with efficacy of post-line ICI treatment was investigated. among those patients whose PD-L1 expression improved after developing EGFR-TKI resistance, survival analysis showed that treated with ICIs had better progression-free survival (PFS) and overall survival (OS) compared with those treated with other treatments (PFS, P < 0.005; OS, P < 0.040, **Figures 4B, C**).

## Discussion

The applicability of ICI-based therapies to patients with EGFR-mutated NSCLC who carry PD-L1 TPS > 50% and progress after EGFR-TKI therapy remains controversial. Our investigations suggest that ICI-based treatment may provide more favorable survival for these patients than classical chemotherapy. ICI combined with chemotherapy seems to be the preferred therapy compared to ICI monotherapy.

Previous studies have shown that patients with advanced NSCLC carrying EGFR mutations have a poor response to immunotherapy, and a possible mechanism for this poor response is the low expression of PD-L1 or the lack of infiltrating T cells in the tumor microenvironment (TME) (11–14). The TME generalization may change with the progression of the tumor, and therefore, resistance to EGFR-TKI may enhance the response to immunotherapy response (7, 15, 16). As reported in the EGFR+/ALK+ cohort in the ATLANTIC study, if PD-L1 expression is greater than 25%, monotherapy with durvalumab led to favorable outcomes with median PFS and OS of 1.9 and 13.3 months, respectively (17).

Previous studies have reported that chemotherapy alone may be the best option when resistance to EGFR-TKI is present (18). In the present study, we compared the outcomes of ICI-based therapy with chemotherapy alone and found that ICI-based treatment had a significant prognostic advantage.

The combination of chemotherapy and immunotherapy enhances the infiltration of effector T cells and downregulates the expression of immunosuppressive cells (19, 20). Ultimately, the efficacy of immunotherapy may be improved. A critical phase II study showed that in EGFR-TKI-resistant NSCLC, ICI combined with chemotherapy resulted in good objective remission rates (ORR, 50%) and survival time (PFS, 7.0 months; OS, 23.5 months) (21). More importantly, a retrospective study also showed the value of ICI combination chemotherapy in metastatic NSCLC after EGFR-TKI resistance (22). In our study, ICI combination therapy resulted in PFS of 10.3 months and OS of 41.6 months in NSCLC patients carrying EGFR mutations and PD-L1 TPS ≥ 50% after developing EGFR-TKI resistance without T790M mutations. The survival time in this study was longer than other studies. The possible reason was that the population included in our study had a higher level of PD-L1 expression than other studies, and NSCLC patients with PD-L1 TPS ≥ 50% seemed to benefit from immunotherapy. Subgroup analysis in our study found that the PFS and OS benefit of IC was significant in most subgroups, except for patients with primary liver metastases and other mutations in EGFR, because the sample was too small to calculate HR and 95% CI.

PD-L1 expression is an effective predictor for ICI response in NSCLC (23). Previous study found that targeted therapy was associated with a significant increase in PD-L1 expression in tumor cells in postprogression tumor samples compared with those obtained at baseline, especially in the case of T790M-negative patients (24). Our reseaech also found that PD-L1 expression was remarkablely improved after receiving EGFR-TKI treatment. Among those patients whoso PD-L1 expression improved after developing EGFR-TKI resistance, survival analysis showed that treated with ICIs had better progression-free survival (PFS) and overall survival (OS) compared with those treated with other treatments, which means improved PD-L1 expression after developing EGFR-TKI resistance may indicate a good response to immunotherapy in poster-line treatment.

Several possible limitations can be seen in our study. First, this study is a retrospective single-center study, which inevitably causes selection bias. Secondly, the lack of sufficient tissue samples for exploratory analysis is a limitation of this study. Therefore, we could only perform PD-L1 status testing on a limited number of specimens before ICI treatment. Multicenter prospective and large sample studies are expected to provide more comprehensive insights into EGFR-mutated NSCLC patients carrying PD-L1 TPS > 50%.

In conclusion, our study suggests that for patients with advanced NSCLC with EGFR mutations and PD-L1 TPS ≥ 50% who have failed prior EGFR-TKI therapies without T790M mutation, ICI-based treatment could provide more favorable survival than classical chemotherapy. More importantly, ICI combination therapy was superior to ICI monotherapy.

## Data availability statement

The sequencing data presented in the study are deposited in the Figshare repository (https://figshare.com/articles/dataset/Patient_genetic_data_xlsx/22664644).

## Ethics statement

The studies involving human participants were reviewed and approved by the Institutional Review Board of the Shanghai Chest Hospital. The patients/participants provided their written informed consent to participate in this study.

## Author contributions

WZ, BH, YQL and YJL: study conceptualization and manuscript revision. YJL, HJ and FQ: paper writing. YC, WSZ and YJL: data analysis and figures. YZ and JL: clinical data collection. WZ, BH and YQL: study progress supervision. All authors listed have made a substantial, direct, and intellectual contribution to the work and approved it for publication. All authors contributed to the article and approved the submitted version.
