# Supplementary figures and images for: Efficacy of ICI-based treatment in advanced NSCLC patients with PD-L1≥50% who developed EGFR-TKI resistance
Source: Front Immunol. 2023 May 17;14:1161718. doi: 10.3389/fimmu.2023.1161718 (PMC10230103; doi:10.3389/fimmu.2023.1161718)

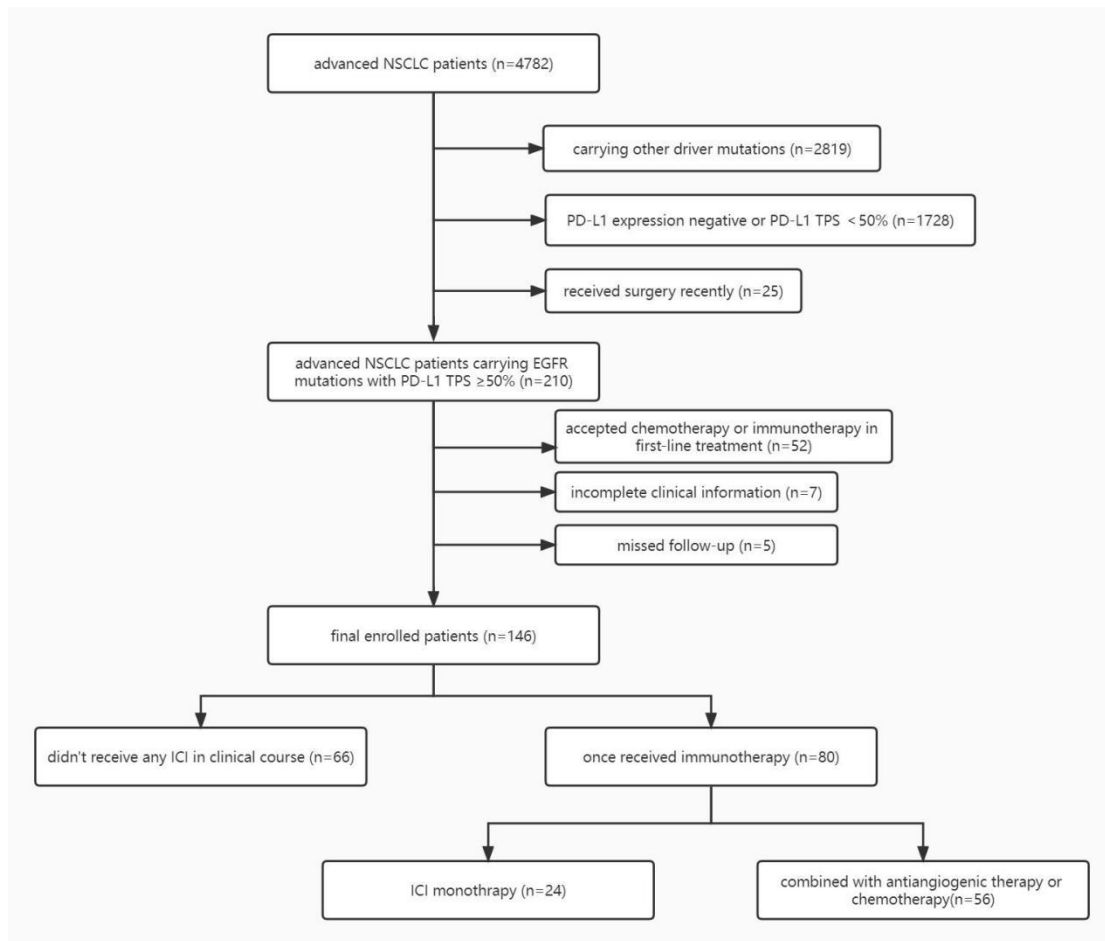

**Supplement Figure 1. Flow chart**

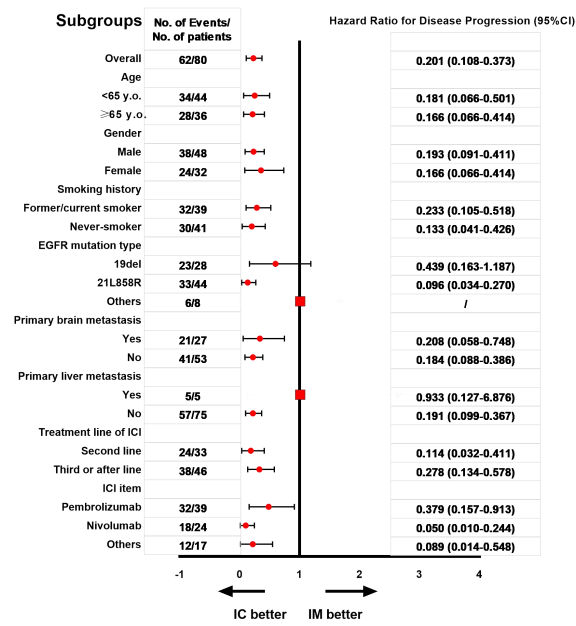

**Supplement figure 2.**

Supplement: Supplementary file 1 [file DataSheet_1.pdf]
